# Validation of a Novel Technique and Evaluation of the Surface Free Energy of Food

**DOI:** 10.3390/foods6040031

**Published:** 2017-04-20

**Authors:** Tugce Senturk Parreidt, Markus Schmid, Carolin Hauser

**Affiliations:** 1 Fraunhofer Institute for Process Engineering and Packaging IVV, Giggenhauser Straße 35, Freising 85354, Germany; carolin.hauser@ivv.fraunhofer.de; 2 Chair for Food Packaging Technology, Technische Universität München, Weihenstephaner Steig 22, Freising 85354, Germany; markus.schmid@ivv.fraunhofer.de

**Keywords:** contact angle, surface energy, edible coating, image processing, drop shape analysis, food

## Abstract

Characterizing the physical properties of a surface is largely dependent on determining the contact angle exhibited by a liquid. Contact angles on the surfaces of rough and irregularly-shaped food samples are difficult to measure using a contact angle meter (goniometer). As a consequence, values for the surface energy and its components can be mismeasured. The aim of this work was to use a novel contact angle measurement method, namely the snake-based ImageJ program, to accurately measure the contact angles of rough and irregular shapes, such as food samples, and so enable more accurate calculation of the surface energy of food materials. In order to validate the novel technique, the contact angles of three different test liquids on four different smooth polymer films were measured using both the ImageJ software with the DropSnake plugin and the widely used contact angle meter. The distributions of the values obtained by the two methods were different. Therefore, the contact angles, surface energies, and polar and dispersive components of plastic films obtained using the ImageJ program and the Drop Shape Analyzer (DSA) were interpreted with the help of simple linear regression analysis. As case studies, the superficial characteristics of strawberry and endive salad epicarp were measured with the ImageJ program and the results were interpreted with the Drop Shape Analyzer equivalent according to our regression models. The data indicated that the ImageJ program can be successfully used for contact angle determination of rough and strongly hydrophobic surfaces, such as strawberry epicarp. However, for the special geometry of droplets on slightly hydrophobic surfaces, such as salad leaves, the program code interpolation part can be altered.

## 1. Introduction

The surface free energy (SFE) is one of the important thermodynamic quantities describing the state of equilibrium of atoms at the surface layer of materials [1]. The term is often used as a measure of the adhesive properties and is a characteristic quantity for each substance. In another definition, the surface free energy is the work necessary for creating a new surface unit, while separating two phases in equilibrium, in a reversible isothermal process [1].

The accurate determination of the SFE is one of the key parameters in controlling a wide range of phenomena, such as the precise characterization of a solid material surface (e.g., hydrophobicity), wettability, and effective spreading of a coating material on a solid surface. These aspects influence many industrial applications in which adhesive properties play an important role, such as adhesion, coating, printing, and lubrication [2,3,4,5,6]. 

Strategies to prevent microbial spoilage and quality loss in fresh foods for a longer period are important for the food industry [7,8,9]. Research on edible films and coatings has been intense in recent years due to these being environmentally friendly and an effective alternative to non-biodegradable plastic packaging [10,11]. Edible films and coatings are any type of material used for enrobing food and produce an extra physical barrier to extend shelf life, improve appearance and maintain the quality of the product [11,12,13]. The films and coatings can be eaten together with food with or without further removal [12]. Edible coatings and films must be designed taking into account the surface properties and wettability of coating emulsions in order to realize effective spreading of a coating solution on a food surface [14,15,16,17]. Studying the surface properties of products is one of the most important steps for effective formulation of edible coatings [17,18]. 

Contact angle measurements can be performed easily on a smooth and flat surface. In contrast, the surface energies of food products are very difficult to measure owing to the surface topography. Due to not having flat and reflective surfaces, differentiating the contact surface of the test liquid on food and determining the contact points of the droplet using a contact angle meter (contact angle goniometer) is usually not possible. The objective of the present study was to use a novel method, namely a snake-based ImageJ program, while presenting an experimental design described in detail to accurately measure the contact angles of rough and irregular shapes, such as food samples and, thus, accurately determine the surface energy of food materials. In order to achieve this goal, the contact angle data using the novel technique were first compared to the contact angle data using a conventional technique, namely using a drop shape analyzer device. Mathematical relationships between the two measurement methods were established.

### Determination of the SFE: Theoretical Information

The surface energy and surface tension of a liquid are identical. Many techniques are available for measuring the surface tension of liquids [19]. However, measuring the surface energy of a solid is not straightforward. Surface energy values can be determined indirectly using various liquids of known surface energy and their components [6]. Drops of a series of liquids are placed on a solid surface and the contact angles are measured. For choosing the set of liquids, specific surface interactions, surface reactivities, and surface solubilities should be taken into consideration [20]. In addition to the lack of universally defined probe liquids, choosing a theory for converting contact angle data into surface energy data is the other critical step. Calculation methods for the surface free energy of solids have been reviewed by Zenkiewicz [5]. Based on the number of components, the most common surface energy theories are the one-component model—Zisman theory; two-component model—Owens/Wendt theory and Fowkes theory; and, lastly, the three-component model—Van Oss theory. 

The contact angle meter used in this study converted the contact angle data into surface energy with the help of one of the most common methods for SFE calculation, the Owens/Wendt theory. Therefore, the same theory was used for the data acquired using contact angle evaluation software to ensure the compatibility of the results.

The Young equation (Equation (1)), which describes the phenomena of thermodynamic wetting (small or zero contact angle, *θ*, between the liquid and solid), is the basis of the method for calculating the SFE from contact angle data:

(1) $$\gamma_{SV}=\gamma_{SL}+\gamma_{LV}\cos\theta$$

Here, $\gamma_{SV}$ is the surface tension on the solid-gas interphase, $\gamma_{SL}$ is the surface tension on the solid-liquid interphase, $\gamma_{LV}$ is the surface tension on the liquid-gas interphase, and *θ* is the equilibrium contact angle [21].

Owens and Wendt developed a two-parameter model—with a dispersive component (*D*) and a polar component (*P*)—for determining the surface energy of a solid, expressed by Equation (2a) and Equation (2b):

(2a) $$\gamma_{L}=\gamma_{L}^{P}+\gamma_{L}^{D}$$

(2b) $$\gamma_{S}=\gamma_{S}^{P}+\gamma_{S}^{D}$$

where γ_S_ is the SFE, γ_S_^P^ is the polar component of the SFE, and γ_S_^D^ is the dispersive component of the SFE. The polar component is the sum of polar, hydrogen, inductive, and acid-base interactions, while the dispersive component accounts for van der Waals and other non-site specific interactions [1,20,21,22].

In their study, Owens and Wendt [21] used Equation (3):

(3) $$\gamma_{SL}=\gamma_{SV}+\gamma_{LV}-2\sqrt{\gamma_{SV}^{D}\gamma_{LV}^{D}}-2\sqrt{\gamma_{SV}^{P}\gamma_{LV}^{P}}$$

Mathematically, the Owens/Wendt theory is based on two fundamental equations (Equation (1) and Equation (3)) In order to derive the solution, converting the equation into a linear fit to incorporate data for more than two liquids is the key [23]. After the necessary rearrangement to get the linear equation (*y* = m*x* + n), the following equation was obtained:

(4) $$\frac{\left(1+cos\theta \right)\;\gamma_{L}}{2\sqrt{\gamma_{L}^{D}}}=\sqrt{\gamma_{S}^{P}}\sqrt{\frac{\gamma_{L}^{P}}{\gamma_{L}^{D}}}+\sqrt{\gamma_{S}^{D}}$$

As is clear from Equation (4), the contact angle values of test liquids on the solid surface and the surface tension components of the liquids must be determined accurately in order to define the polar and dispersive components and, therefore, the surface free energy of a solid material. In other words, being able to determine the contact points of the test liquids on the solid and, hence, accurately measure the angles is the crucial step for determining the surface hydrophobicity. Determination of the contact points of test liquids on food samples using a conventional contact angle meter/goniometer is, however, not possible. The main aim of the present study was, therefore, to present an experimental procedure using a novel method for measuring the contact angles and surface free energies of rough and irregular-shaped food samples.

## 2. Materials and Methods 

### 2.1. Materials

Validation of the contact angle measurement process was carried out on four different plastic films: polytetrafluoroethylene (PTFE) (thickness: 500 µm, SAHLBERG GmbH and Co. KG, Feldkirchen, Germany), biaxially-oriented polypropylene (BOPP) (thickness: 20 µm, Taghleef Industries GmbH, Holzhausen an der Heide, Germany), polyethylene terephthalate (PET-160 μm) (thickness: 160 μm, GEBA GmbH, Seewald, Germany), and polyethylene terephthalate (PET-12μm) (thickness: 12 μm, Mitsubishi Polyester Film GmbH, Wiesbaden, Germany). These plastic films were carefully selected to ensure a wide range of surface tension values. 

Various test liquids, namely water for chromatography (Merck KGaA, Darmstadt, Germany), diiodomethane (Sigma-Aldrich, St. Louis, MO, USA), ethylene glycol (Sigma-Aldrich, St. Louis, MO, USA), and glycerol (Sigma-Aldrich, St. Louis, MO, USA), were used for the surface energy measurements. 

Fresh endive salad *(Cichorium endivia)* and strawberries (*Fragaria ananassa*) were purchased from a local market (Freising, Germany). Samples were carefully selected to ensure uniformity of color, ripeness, and physical appearance based on visual analysis. Only the green parts of the endive salad leaves were used for the contact angle measurements. Before the measurements, the samples were left at ambient temperature (20 ± 1 °C). The samples (~3 cm × 2 cm) were cut into rectangular shapes. 

### 2.2. Methods

#### 2.2.1. Surface Tension of the Test Liquids

The surface tension of the test liquids (water, diiodomethane, ethylene glycol, and glycerol) and its polar and dispersive components were determined using the pendant drop method with a drop shape analyzer (DSA1 v1.90, Kruss GmBH, Hamburg, Germany) [24]. 

#### 2.2.2. Validation of the Novel Method

The surface energy measurement of the four different plastic films was carried out by the contact angle (CA) technique with two different methods: The drop shape analyzer (DSA) (DSA1 v1.90, Kruss GmbH, Hamburg, Germany) and the ImageJ program with the DropSnake plugin [25].

In the first part of the analysis, both the CA (*θ*) and surface energy (γ_S_) of the plastic films were determined with the DSA. Droplets of the test liquids (water, diiodomethane, ethylene glycol) were automatically placed with a 500 μL syringe (Hamilton, Switzerland) and 1.991 mm needle (Kruss GmbH, Hamburg, Germany). The device performed the CA measurement by the sessile drop technique and γ_S_ calculation using the Owens and Wendt (1969), Rabel (1971), and Kaelble (1970) theory [20].

In the second part of the study, photographs of droplets on the surfaces were taken with a Canon EOS 600D digital camera (Canon, Japan) fitted with auto extension tube set (Kenko, Japan). The photographs were taken in a dark room. Two yellow-colored light sources (35 W, 50/60 Hz, 220–240 V, 5400 K) were placed on two sides of the sample. The distances between the light sources and the samples and between the camera and the samples were fixed for all measurements and were 17 cm and 7 cm, respectively. At ambient temperature (20 ± 1 °C), small droplets (3 μL) of the test liquids (water, diiodomethane, ethylene glycol) were manually placed using a micropipette on the surface of the plastic films, which were fixed with adhesive tape so that horizontal, flat surfaces were obtained. As drop deposition has been found to be a critical step in previous work [8], the droplets were dispersed gently keeping the micropipette perpendicular to the surface. To avoid changes in the shape of the droplets due to gravitational force, the time between surface-liquid contact and photographic exposure was never longer than 7 s. 

The contact angles at the left and the right margins of the droplet were determined using ImageJ software [25] with the DropSnake plugin [26]. This plugin was ideal for measuring asymmetric drops because no shape assumptions were used. Seven knots were manually placed along the contour of the drop and the contact angle was obtained by a polynomial fit [27]. The CA values of the drop were automatically calculated based on this fit. The surface energies of the plastic films were calculated by the theory of Owen/Wendt in Microsoft Excel 2010 (Microsoft Corp., Redmond, WA, USA) in order to achieve the compatibility of the two measuring methods.

#### 2.2.3. Case Studies

The experimental design described in Section 2.2.2, was also used for determination of the surface free energy and its polar and dispersive components. Three microliter droplets of water, diiodomethane, and ethylene glycol were carefully placed on strawberries. However, glycerol was used instead of ethylene glycol for the endive salad experiments in order to have more apparent contact angles. The contact angle values of the droplets were determined using the ImageJ program with the DropSnake plugin with the help of high-resolution photos.

#### 2.2.4. Statistical Evaluation

Fifteen measurements were performed for each condition (*n* = 15). The mean, standard deviation, regression analysis, fitting performance, and graphics were performed using R 3.3.2 for Windows with the packages ggplot2 [28], gridExtra [29], ggrepel [30], car [31], and lsr [32]. The R^2^ statistic (coefficient of determination) and graphical residual analysis were used to evaluate the model fit. 

## 3. Results

### 3.1. Surface Tension of the Test Liquids

The surface tension and the polar and dispersive forces of the test liquids are given in Table 1. The test liquids were measured periodically in order to track changes in their values. Throughout the experiments, no significant changes (>0.1 mN/m) were observed in the values indicated in Table 1. Therefore, the surface tension and component values are not specified with standard deviations. Water for chromatography, diiodomethane, ethylene glycol, and glycerol were chosen as the test liquids in order to span the whole range of fluids from dispersive to polar. 

### 3.2. Validation of the Snake-Based Method

#### 3.2.1. Contact Angle Measurement

**Figure 1:**
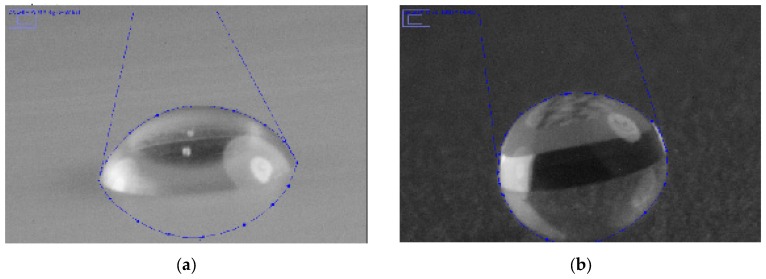
ImageJ program screenshots of: (**a**) contact angle measurement of diiodomethane on PTFE; and (**b**) contact angle measurement of water on PET-160 µm.

The hydrophobicity and/or hydrophilicity of a surface can be specified by contact angle measurement. As recommended by Nguyen and Johns [33], pure liquids were selected according to two characteristics: their ability to form droplets on the surface (γ_L_ > γ_C_, critical surface tension of the plastic films) and known dispersive and polar components. One non-polar liquid (diiodomethane) and two polar liquids (water and ethylene glycol) constitute a good set for CA and SFE measurements. Figure 1 shows screenshots of contact angle measurement of plastic films using the computer-based ImageJ program. 

**Figure 2:**
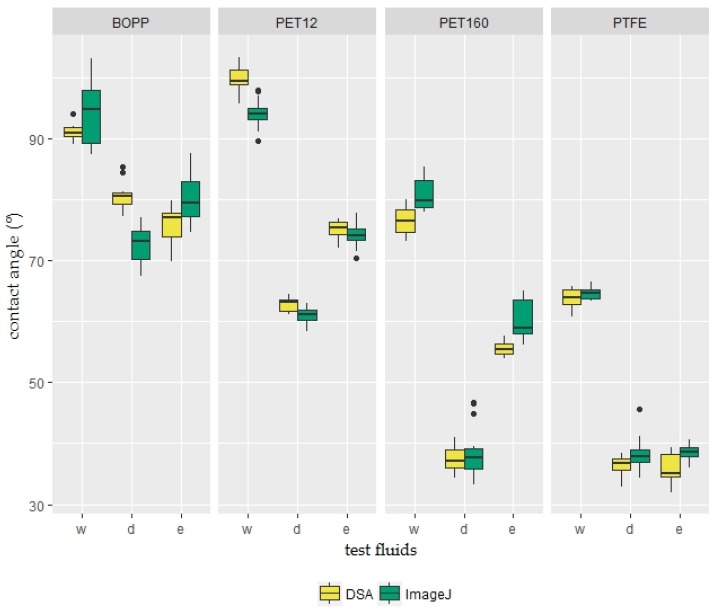
Box plot showing the variation of the contact angle of the test liquids (**w**: water; **d**: diiodomethane; **e**: ethylene glycol) using two different measurement methods (DSA and ImageJ) on plastic films (n = 15).

The contact angle results and distribution characteristics of three test liquids (water, diiodomethane, and ethylene glycol) on PTFE, BOPP, PET-160 μm, and PET-12 μm surfaces are presented in a box plot diagram (Figure 2). Lines across the boxes indicate the medians and each outlier outside the whiskers is represented by an individual mark. The minimum and maximum values in the dataset are used as end points for the whiskers. The contact angle results of the two methods show the same increasing-decreasing pattern (θ_water_ > θ_e.glycol_ ≥ θ_d.methane_). However, the differences in the medians and distributions of the two methods are worthy of further investigation. These results demonstrate that the ImageJ program can be used as an alternative to DSA systems. However, the correlation between the results of the DSA system and ImageJ software should be expressed mathematically in terms of compatibility and reliability. 

#### 3.2.2. Surface Free Energy Measurement by the Polar—Dispersive Approach

**Figure 3:**
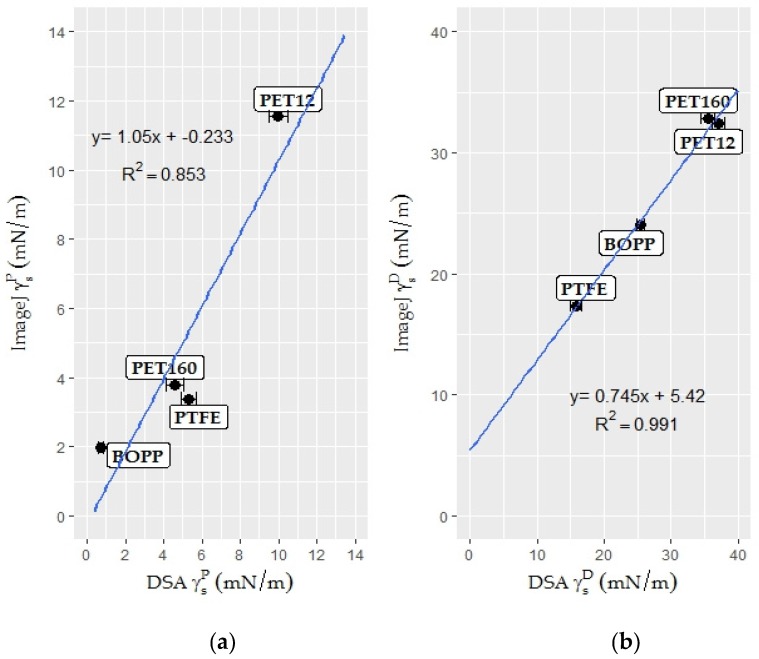
Scatterplots: (**a**) polar (γ_S_^P^) surface energy component of the novel method plotted as a function of the DSA results in mN/m for plastic films (*n* = 15); and (**b**) dispersive (γ_S_^D^) surface energy component of the ImageJ results plotted as a function of the DSA results in mN/m for plastic films (*n* = 15).

**Figure 4:**
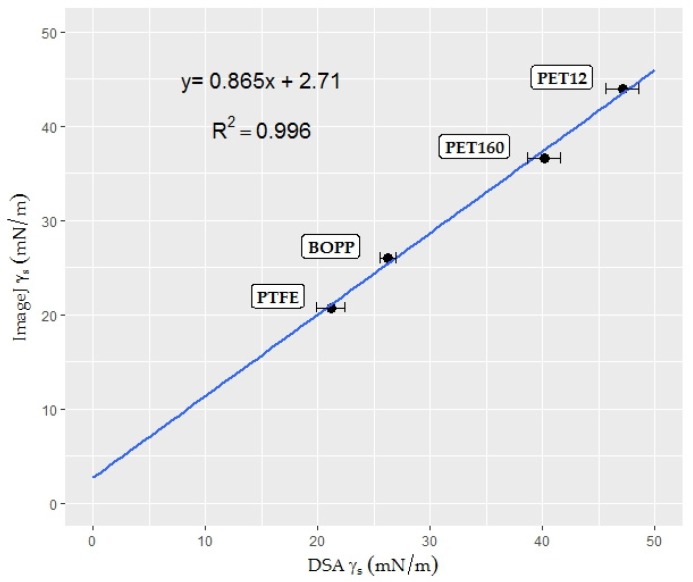
Scatterplot of the surface free energy (σ_s_) results of the ImageJ program plotted as a function of the DSA results in mN/m for plastic films (*n* = 15).

The surface energy of a solid cannot be directly measured; values must be calculated from various liquid-solid contact angles using well-known theories. In the present study, the Owen/Wendt method was used in order to achieve compatibility between the two measuring methods. The relationships between the polar components (Figure 3a), dispersive components (Figure 3b), and surface energies (Figure 4) are illustrated in the graphs. Error bars, showing standard deviations, were only generated for the DSA results. The reason for this is that only one result for each surface energy component can be obtained from the linear regression plot of the Owens/Wendt theory with the aid of Equation (4). The total surface energy is the sum of the polar and dispersive components [19]. 

The graphs suggest that the results obtained using the novel method change linearly with the results of the conventional DSA. Therefore, simple linear regression models allowed us to study the relationship between these two measurement methods. 

The scatterplots show a fairly strong and reasonably linear relationship between the two variables. All of the models, visually, gave a good fit of the data. The validity of the models was checked with both graphical residual analysis and numerical *R*^2^ statistics (coefficient of determination). The *R*^2^ values were found to be high (*R*^2^ > 0.85) for all datasets. Moreover, residuals appeared to behave randomly in generated residual graphs, suggesting that linear regression models fit the data well. The method validation confirms that with the aid of a defined mathematical relationship the novel contact angle measurement tool is suitable for determining the SFE of rough and irregular surfaces, such as the surfaces of fruit and vegetables. The models were, therefore, used for determining the SFE of food products.

### 3.3. Case Studies

#### 3.3.1. Strawberry

**Figure 5:**
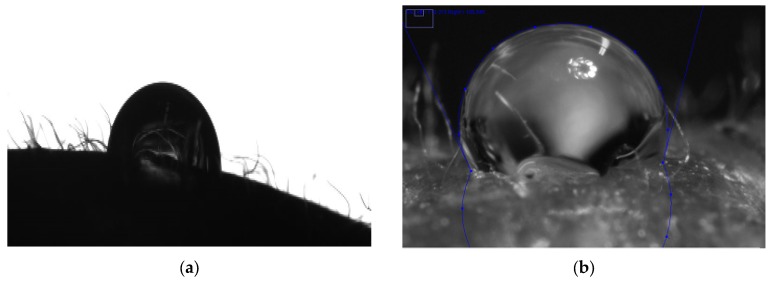
Screenshot photos of 3 µL water droplets on strawberry epicarp: (**a**) taken with DSA system; and (**b**) taken with a digital camera in the drop shape evaluation process.

The surface energy of the strawberry epicarp has been described in the literature before [7]. However, CA measurements were performed using a face contact angle meter. Due to the insufficient light source for the angle meters and rough, pitted, and round food samples, defining the contact points of the liquids on the strawberry surfaces was not easy. Figure 5 shows screenshot photos taken with the DSA system and a digital camera as part of the drop shape evaluation process. Accurately estimation of the angles of the contact points of the test liquids is extremely difficult using the widely-used contact angle meter. However, with the help of more powerful and portable light sources, angles could be determined in the photos taken with a high-zoom camera. 

The special geometry of strawberry reveals the necessity of paying particular attention to some critical details when taking the photos and the calculation processes. The camera was aligned perpendicular to have a horizontal surface of the substrate. The droplets were placed carefully away from seed (achene) holes. However, due to the irregular, convex shape of the strawberry, the surface had small angle slopes (maximum of 10°) in some cases. However the effect which caused by gravitational force was calculated and found to be very small and negligible. Furthermore, the photographs were carefully selected and both left and right contact points of the droplets on the surface were clearly visible.

**Figure 6:**
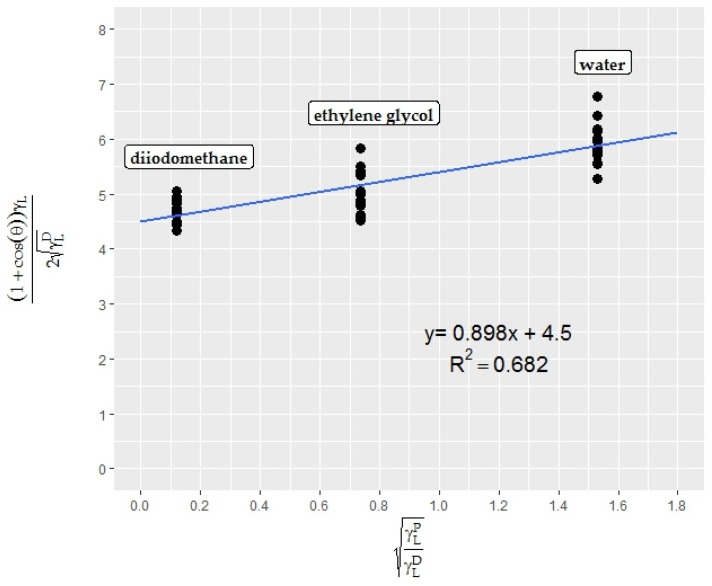
Owens-Wendt plot for strawberry epicarp (*n* = 15).

When considering the attractive forces on a strawberry surface, the contact angle data of test liquids on the surface and the surface tension values (overall, polar, dispersive) of the liquids have to be known and plotted in an Owens-Wendt graph (linear Equation (4)). The CA of water, diiodomethane, and ethylene glycol on strawberry skin was found to be 103.7 ± 2.8, 71.8 ± 3.6, and 80.5 ± 5.7, respectively. No mild or extreme outliers were found in the datasets. The resultant Owens-Wendt plot is shown in Figure 6. 

The superficial characteristics of strawberries are not uniform through the surface. Different parts of the fruit exhibit different surface free energy. Therefore, droplets of the same liquid have different contact angles on different parts of the fruit. In the present study, the contact angles from different parts of different strawberries were plotted in order to denote the entire surface. Nevertheless, using the whole surface led to lower *R*^2^ results. 

The superficial properties of strawberry epicarp were calculated with the help of the equation (*y* = 0.8978*x* + 4.5041) obtained from the Owens-Wendt plot for strawberry epicarp (Figure 6). According to the calculations, the strawberry surface is a low energy surface with a surface free energy of 21.10 mN/m, and polar and dispersive components of 0.81 and 20.29 mN/m, respectively. The program results were also converted into widely-used contact angle meter (DSA) results with the help of linear regression models obtained from Figure 3a,b, and Figure 4 and are presented in Table 2. 

#### 3.3.2. Endive Salad

**Figure 7:**
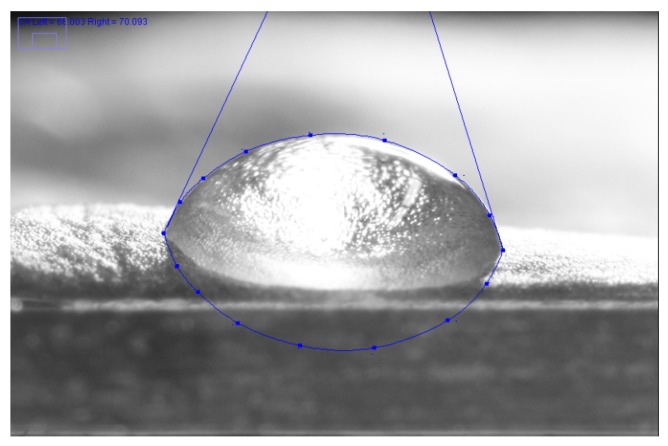
Screenshot photo of a 3 µL diiodomethane droplet on the endive salad surface taken with a digital camera as part of the drop shape evaluation process.

The CA of water, diiodomethane, and glycerol on endive salad was found to be 55.65 ± 9.61, 61.40 ± 5.87, and 58.34 ± 5.14, respectively (for a screenshot see Figure 7). Glycerol was used instead of ethylene glycol in order to have higher and, therefore, more apparent contact angles on endive leaves.

**Figure 8:**
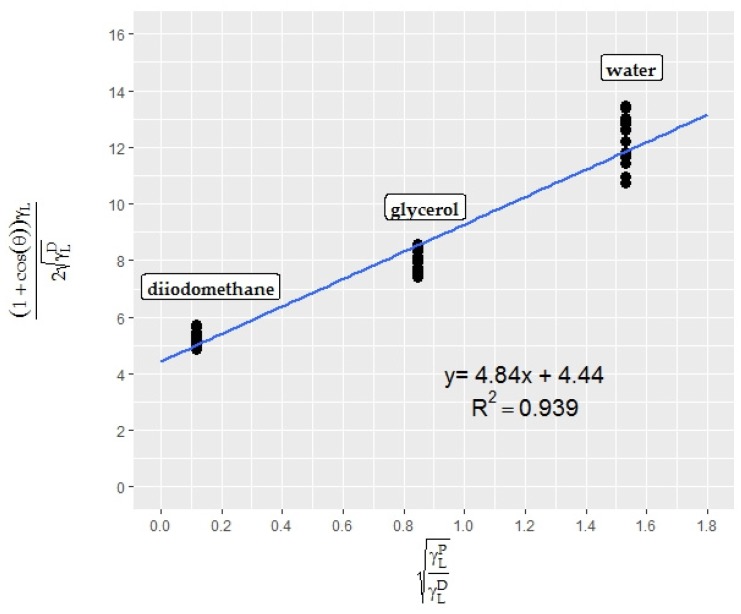
Owens/Wendt plot for endive salad epicarp (*n* = 15).

No mild or extreme outliers were found in the datasets. The resultant Owens-Wendt plot is shown in Figure 8.

The superficial properties of endive leaves were calculated with the help of the equation (*y* = 4.8358*x* + 4.4407) obtained from the Owens-Wendt plot for endive salad. According to the calculations, endive salad has a relatively high energy surface (43.11 mN/m) with polar and dispersive components of 23.39 and 19.72 mN/m, respectively. The results of the program were also converted into widely-used contact angle meter (DSA) results with the help of linear regression models obtained from Figure 3a, b and Figure 4 and are presented in Table 3. 

## 4. Discussion

Most fruit and leafy vegetables that exhibit strong hydrophobicity have surface roughness with microstructures and nanostructures comprising unwettable wax crystals [34]. In the present study two different food materials were carefully selected for their irregular, rough shape. Due to their round shape, pitted structure with seeds (achenes), and hairs attached to the achenes, strawberries are a challenge for accurate contact angle measurement. Similarly, due to the wavy, curly shape of the leaves, surface energy characterization of endive salad is also challenging. A novel method using the ImageJ program with the DropSnake plugin was used to define contact angles for non-axissymmetric drops from digital photographs with high resolution. As a first step, the novel method was validated with the aid of four plastic films, comparing the measured contact angles with those obtained using the widely-used DSA device. The measured SFE with its polar and dispersive components are summarized in Table 4 along with the literature data. The results from this study agree well with those in the literature. The values of the surface free energy and its components increased with the decreasing thickness of the PET film. 

**Figure 9:**
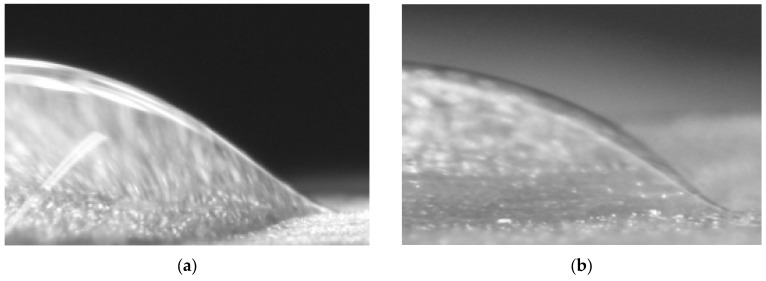
(**a**) Bell-shaped water droplet on an endive salad leaf and (**b**) bell-shaped glycerol droplet on an endive leaf.

With the help of linear regression models, mathematical relationships were determined between the results of two different measurement techniques. However, the dispersive forces which were determined using the ImageJ program were biased compared to the DSA results. The difference between the results may originate from the contact angle interpolation skim of the program. Figure 9 shows the unusual, bell shapes of sessile drops of water and glycerol on endive salad surfaces. In the study of Kako et al., bell-shaped droplets on hydrophilic/hydrophobic surfaces were well documented [38]. As the contact angle results are obtained by a piecewise polynomial fit of the program [26], further research is needed to improve the curve fitting for bell-shaped droplets. 

The surface free energies of strawberry and endive salad surfaces were determined with the help of high-accuracy contact angles. The results of the present study showed that the strawberry surface is a low energy surface (21.26 mN/m) with high dispersive forces (19.96 mN/m) and very low polar forces (0.99 mN/m). These results agree with the findings of Ribeiro et al. who found the surface tension of strawberries to be 28.94 mN/m, with polar and dispersive components of 5.95 and 22.99 mN/m, respectively [7]. On the other hand, Velasquez et al. measured surface energy values of strawberry samples using the acid-base approach and found a value of ~40 mJ/m^2^ (40 mN/m) [39]. The difference between the results may be due to differences in the methods used for surface free energy calculation. 

High dispersive forces with low or zero polar forces indicate that the surface is strongly hydrophobic, meaning that polar liquids, such as water-based coatings, cannot uniformly spread and wet the surface [22,40,41]. It is clear that high solid surface free energy and low liquid surface free energy favors wettability [21]. Therefore, different formulations of edible coatings with effective surfactants should be evaluated for higher wettability of the strawberry surface. 

The surface energy of endive salad, which has not been previously described in the literature, was found to be 43.11 mN/m. However, the higher polar (23.39 mN/m) and dispersive (19.72 mN/m) components indicate that higher surface wettability can be achieved with coatings having a relatively high surface tension. Although, as in the strawberry study, different parts of different endive salad leaves were used for the contact angle measurements, the contact angle results did not show drastic scatter (unlike the results for strawberries), implying the green endive salad leaves have a more uniform surface structure. 

## 5. Conclusions

In the present work, accurate contact angles of test liquids on rough-shaped food samples and, hence, actual surface free energy values, were measured. The novel method that was used was validated by comparing the data with the results obtained using a contact angle meter. The latter is frequently used for flat surfaces in the literature and for industrial applications. Linear regression models and linear equations were defined to represent the mathematical relationship between the two measurement methods. This part of the work will be particularly useful for comparing results obtained using these two measurement methods in future studies.

Strawberries and endive salad were chosen due to their rough and irregular shapes. The surface energies, as well as the polar and dispersive components, were determined using the more accurate contact angles. According to the results, strawberries have a low energy surface (21.26 mN/m) with strong hydrophobicity (high dispersive forces (19.96 mN/m) and very low polar forces (0.99 mN/m)). Effective surface active agents should be added to coating formulations to achieve higher wettability. In contrast, effective spreading of coating solutions on salad surface is relatively easy due to the higher polar (23.39 mN/m) and dispersive (19.72 mN/m) components.

The results show that the ImageJ program with the snake-based approach is an important tool for contact angle measurement of irregular and rough food surfaces. The concept presented in this paper can be used as a guideline for designing coating materials with improved surface wettability, with the help of more accurate contact angle measurements. The program can be developed further to prevent biased data and to obtain better polynomial fitting for bell shaped droplets.

## Figures and Tables

**Table 1:** Surface tension values of the test liquids used in this study in mN/m (T = 20 °C).

Liquids	Surface Tension ($\gamma_{L})$	Polar Forces ($\gamma_{LV}^{\;\;P})$	Dispersive Forces ($\gamma_{LV}^{\;\;D})$
water	72.8	51.0	21.8
ethylene glycol	47.7	16.8	30.9
diiodomethane	50.8	0.7	50.1
glycerol	63.4	26.4	37.0

**Table 2:** Superficial properties of strawberry epicarp. *y*: results obtained with the novel method, linear regression models obtained from Figure 3a,b and Figure 4, and *x*: results calculated using the drop shape analyzer.

Superficial Properties	Novel Method (mN/m)	Linear Regression Models	DSA (mN/m)
Surface free energy	21.10	y=0.865x+2.71	21.26
Polar component	0.81	y=1.05x−0.233	0.99
Dispersive component	20.29	y=0.745x+5.42	19.96

**Table 3:** Superficial properties of endive salad epicarp. *y*: results obtained with the novel method, linear regression models obtained from Figure 3a,b and Figure 4, and *x*: results calculated using the drop shape analyzer.

Superficial Properties	Novel Method (mN/m)	Linear Regression Models	DSA (mN/m)
Surface free energy	43.11	y=0.865x+2.71	46.71
Polar component	23.39	y=1.05x−0.233	22.50
Dispersive component	19.72	y=0.745x+5.42	19.19

**Table 4:** Summary of the SFE and its polar and dispersive components measured on plastic films in the present study along with literature data in mN/m.

Study	Plastic Film	Polar Component (γ_S_^P^)	Dispersive Component (γ_S_^D^)	SFE (γ_S_)
Present study	BOPP	0.71	24.03	26.23
Guimond et al. [35]	BOPP	~0.5 ^1^	~24.5 ^1^	~25 ^1^
Present study	12 µm PET	9.98	37.12	47.10
Present study	160 µm PET	4.58	35.55	40.13
Wang et al. [36]	10 µm PET	-	-	45.6 ^1^
Present study	PTFE	5.31	15.82	21.13
Jun and Qunji et al. [37]	PTFE	3.93	28.42	-

^1^ Units were converted to mN/m.
